# Prospective Cohort Study of Emergency Department Visit Frequency and Diagnoses Before and During COVID-19 Pandemic in Urban, Low-Income, US- and Foreign-Born Mothers in Boston, MA

**DOI:** 10.5811/westjem.59639

**Published:** 2023-11-08

**Authors:** Valerie Osula, Serena Rusk, Lingxin Hao, Bhakti Hansoti, Alison Gemmill, Xiumei Hong, Guoying Wang, Colleen Pearson, William G. Adams, Xiaobin Wang

**Affiliations:** * Johns Hopkins University School of Medicine, Department of Emergency Medicine, Baltimore, Maryland; † Johns Hopkins Bloomberg School of Public Health, Center on the Early Life Origins of Disease, Department of Population, Family and Reproductive Health, Baltimore, Maryland; ‡ Johns Hopkins University, Department of Sociology, Baltimore, Maryland; § Johns Hopkins Bloomberg School of Public Health, Department of International Health, Baltimore, Maryland; ∥ Johns Hopkins Bloomberg School of Public Health, Department of Population, Family and Reproductive Health, Baltimore, Maryland; ¶ Boston University School of Medicine, Boston, Massachusetts; # Boston Medical Center, Department of Pediatrics, Boston, Massachusetts; ** Johns Hopkins University School of Medicine, Department of Pediatrics, Baltimore, Maryland

## Abstract

**Background:**

The coronavirus 2019 (COVID-19) pandemic fundamentally changed how populations interface with the healthcare system. Despite historical spikes in US mortality during the pandemic, emergency department (ED) visits were paradoxically low. This is a concerning phenomenon that raises a red flag regarding access to care, especially among vulnerable populations. In this study we sought to understand how ED utilization evolved during the COVID-19 pandemic among traditionally understudied, low-income, racially diverse US- and foreign-born mothers.

**Methods:**

This is a secondary analysis of a pre-existing dataset of 3,073 participants enrolled in the Boston Birth Cohort at birth and followed prospectively. We obtained ED visit diagnoses from 2019 and 2020 via electronic health records, categorized according to the International Classification of Diseases, 10^th^ Revision, and compared them using graph plots, chi-square, and negative binomial regression.

**Results:**

The number of ED visits decreased by 29.1% (*P* < 0.001) from 2019 (1,376) to 2020 (976). However, visits for infectious and parasitic diseases, including COVID-19, increased by 90.6% (32:61) with COVID-19 accounting for 77% of those visits in 2020 (47/61). Mental health-related visits increased by 40.9% (44:62), with diagnoses of alcohol use disorder increasing by 183% (6:17). Regression analysis showed 50% less ED utilization among foreign- vs US-born participants; however, the increase in infectious diseases visits was greater among foreign-born compared to US-born mothers (185% vs 26%, *P* = 0.01), while the increase in mental health diagnoses was greater among US-born mothers (69% vs −33%, *P* = 0.10).

**Conclusion:**

Despite a decrease in total ED visits during the pandemic, there was an increase in COVID-19- (immigrant > US born) and mental health- (US-born only) related visits. Our findings demonstrate that EDs remain a critical access point for care for minority populations and have implications for preparedness, resources, and services of EDs in urban settings to better address the needs of communities. However, alternative avenues for healthcare services for these populations, particularly during health crises, warrant further investigation.

Population Health Research Capsule What do we already know about this issue?

*The early COVID-19 pandemic, which disproportionately affected people of color, was associated with significant mortality and decreased ED utilization.*

What was the research question?

*How did ED utilization patterns among minority mothers change during the COVID-19 pandemic?*

What was the major finding of the study?

*Despite a 29% decrease in overall ED visits, there was a 41% increase in ED mental health visits; diagnoses of alcohol use disorder increased by 183% (P = 0.003).*

How does this improve population health?

*The increased psychological burden associated with COVID-19 among minority mothers highlights the need to expand supportive services for this population.*

## INTRODUCTION

Coronavirus 2019 (COVID-19) disease has caused significant morbidity and mortality; according to the World Health Organization (WHO), there have been over 91 million reported cases of COVID-19 with over a million deaths.^1^ The pandemic affected not only physical health but had a profound effect on mental health, social interactions, education, economic growth, and overall well-being.^2^ ^–^ ^5^ Prior literature has shown that COVID-19 impacted how communities interface with the healthcare system; specifically, one study in Minnesota demonstrated a 49.3% decline in emergency department (ED) visits,^3^ while another in Germany demonstrated a 63.8% decrease in ED pediatric visits^6^ as a result of the COVID-19 pandemic.

Despite this decrease in ED utilization, people still have healthcare needs, particularly for mental health services. More recent evidence found an increase in outpatient adult mental health visits,^7^ particularly among low-income and urban populations^8^; however, there is limited evidence of a similar pattern in large-scale studies or ED settings. A study by Rusk et al on pediatric utilization within a similar population also showed an increase in outpatient visits for mental health despite an overall decrease in other kinds of outpatient healthcare encounters.^9^

While emerging studies highlight the impact of COVID-19 on mental health,^2^ ^,^ ^10^ less is known regarding the mental health implications of COVID-19 as it relates to ED presentations, particularly among people of color, immigrants, and those of lower socioeconomic status who were disproportionately affected by the COVID-19 pandemic.^11^ ^,^ ^12^ Black and Hispanic populations are more likely to be infected with and die from COVID-19 infection.^11^ ^–^ ^14^ Similar trends have been observed as it relates to mental health: Research has shown higher rates of psychological stress and substance abuse disorders among minority populations in relation to the COVID-19 pandemic.^15^ ^–^ ^17^ These findings were largely based on survey data and data from non-ED settings; thus, it is important that we study trends among this population to improve their health outcomes. Additionally, prior studies have documented lower rates of psychological disorders and substance use disorder among those born outside of the US^18^ ^,^ ^19^; however, data is limited as it relates to ED populations and presentations for mental health-related complaints.

While recent studies have looked at changes to ED visit patterns around the world, there is a paucity of data examining both the trends and reasons for changes in ED visits as it relates to maternal and mental health, especially among low-income minority, immigrant populations. In this study we aimed to evaluate changes in both the numbers and diagnoses for ED visits, particularly for mental health-related visits, during the COVID-19 pandemic (2020) compared to baseline (2019) among a sample of US- and foreign-born, urban, low-income, racially diverse, underrepresented mothers.

## METHODS

### Study Design

Participants for this study came from the Boston Birth Cohort (BBC), which was initiated in 1988 in response to rising pre-term birth rates in the US particularly among minority populations. This ongoing birth cohort enrolls mother-child dyads shortly after delivery at Boston Medical Center (BMC). Mothers and their children who continue to receive medical care at BMC were invited to participate in the postnatal follow-up study, which includes electronic health record (EHR) surveillance. Further details regarding recruitment have been published in a profile of the BBC.^20^

### Boston Birth Cohort

The full BBC follow-up cohort is comprised of 3,073 racially diverse and primarily low-income women who continue to receive care at BMC and consented to postnatal follow-up.^8^ This cohort included a robust dataset to track ED utilization among a diverse population and, thus, was the focus of our study. In this study we analyzed ED utilization among the 796 women who visited the ED in 2019 and/or 2020. This created two datasets for analysis. The first dataset is comprised of ED encounters from 2019 and 2020; the observations are ED encounters. The second dataset contains demographic information on participants, where observations correspond to individuals. For the primary analysis we used the ED encounters dataset. The second dataset with demographic information was used to provide additional context about participants. The BBC received institutional review board approval from both Johns Hopkins Bloomberg School of Public Health and BMC. For clarification, the term “women” in this study refers specifically only to women who have given birth to children as defined in our cohort and for the remainder of the paper will be referred to as “mothers.”

### Data Collection

Enrollment into the BBC is performed at BMC by research assistants. Eligible mothers are approached and consented within 1–3 days postpartum, and a baseline questionnaire interview is administered. Mothers and children beginning at six months old are invited to consent to enrollment in the longitudinal, follow-up study, which allows for EHR surveillance. The BMC field team extracts relevant clinical data and diagnoses for the follow-up study from the Clinical Data Warehouse (CDW) at BMC. For our study, the CDW was then queried by field directors for ED visits and filtered by year. We then collated this data into International Classification of Diseases, 10^th^ Rev, Clinical Modification (ICD-10-CM) system categories, which we used for our study analysis. Further details on data collection have been previously published.^20^

We evaluated all ED visits among study mothers from January to December of 2019 and 2020. Information regarding visit diagnoses was collected from the BMC CDW, which holds the EHR for research. All maternal ED visits resulting in at least one diagnosis in 2019 (before the pandemic) and 2020 (intra-pandemic period) were analyzed. We excluded visits where participants “eloped,” “left without being seen,” or did not receive a diagnosis. Each encounter resulted in at least one visit diagnosis with a maximum of three, and there were secondary and tertiary diagnoses in 50% and 25% of ED encounters, respectively (Supplemental Table 1 and 2). Secondary and tertiary diagnoses were most likely representative of additional or incidental diagnoses. Upon examination of secondary diagnoses, 50% were categorically similar to the primary diagnosis; thus, we chose to use the primary diagnosis alone for analysis as this was present for all participants and would represent the most pertinent reason for the ED visit.

### Statistical Analysis

**Figure 1.**
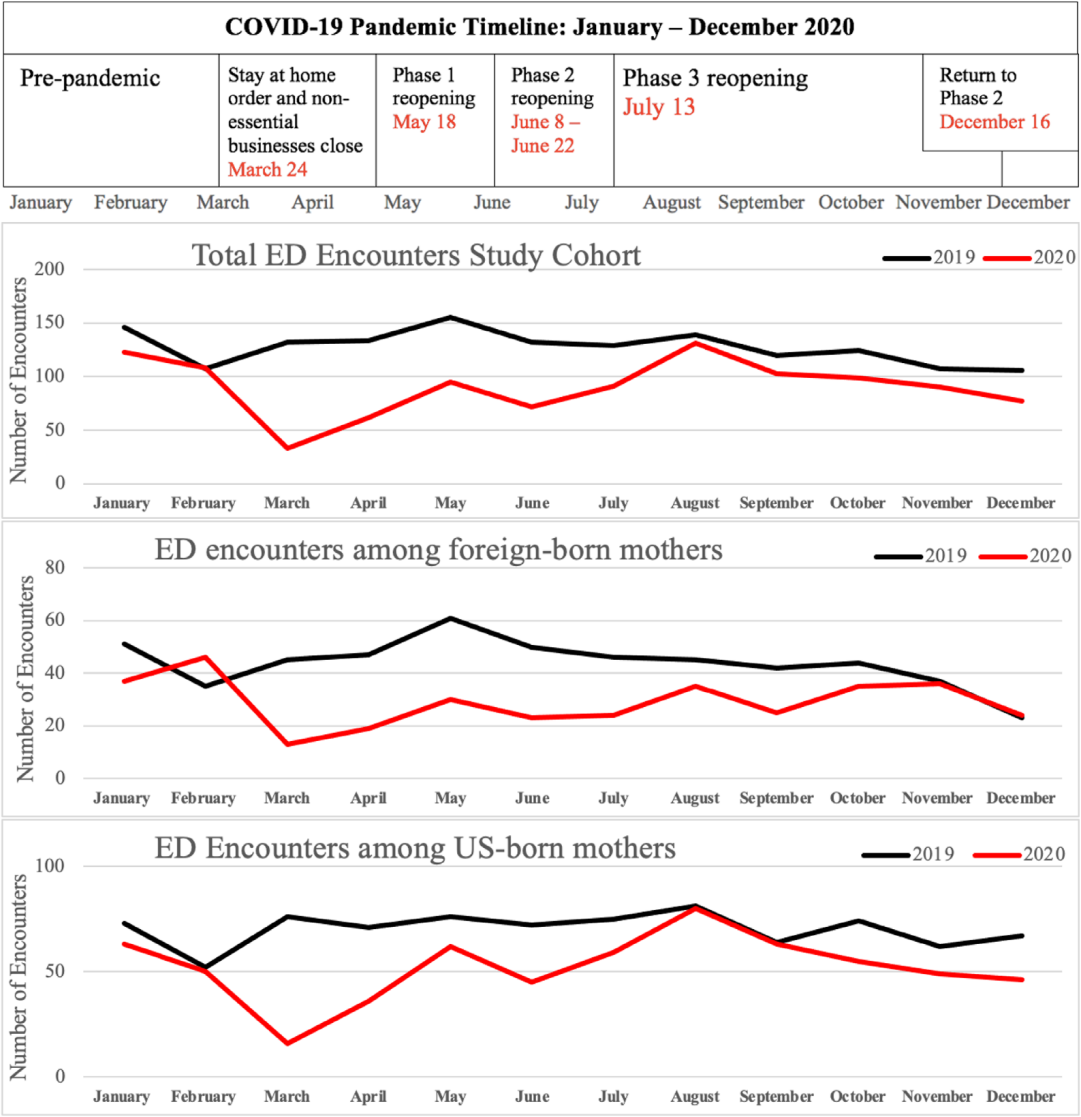
The number of monthly emergency department visits among foreign-born and US-born mothers in the study sample from the full Boston Birth Cohort before and during COVID-19. *ED*, emergency department.

We performed sensitivity analysis of perinatal characteristics of mothers (Table 1). The total number of ED visits for the entire study cohort, as well as by nativity (eg, US- and foreign-born mothers) is shown in Table 2, and we calculated percentage change from 2019 to 2020 (Table 3). Data is also presented as a line graph to reflect the longitudinal patterns for 2019 and 2020, respectively (Figure 1). To understand how pre- and intra-pandemic periods and participant demographics predict the count of ED visits, we fit two negative binomial regression models to our full sample data. Model 1 includes the following: a pandemic period indicator; foreign-born, race/ethnicity (Hispanic and non-Hispanic White, Black and other, with non-Hispanic White as the reference); level of education (lower than high school, high school, with college-educated as the reference); low family income level (lower than $65,000 vs otherwise); and age of delivery <35 years. Model 2 added an interaction term between immigrant status and the pandemic indicator. The coefficients, standard errors, and significance levels for both Model 1 and Model 2 are represented in Supplemental Table 3.

**Table 1.** Summary of preconception and perinatal characteristics of the participating mothers in total sample and by maternal place of birth.

		ED users in 2019 and/or 2020			
Characteristic (% or mean [SD])	Full maternal BBC cohort	Study sample ED users in 2019 and/or 2020	Mothers born outside US	Mothers born in US	p-value^1^
Total n	3,073	796	407	382	
Maternal demographic characteristics					
Maternal race					***
Non-Hispanic White	232 (7.5)	34 (4.3)	4 (1.0)	29 (7.6)	
Non-Hispanic Black	1,786 (58.1)	514 (64.6)	224 (55.0)	286 (74.9)	
Hispanic	686 (22.3)	152 (19.1)	112 (27.5)	39 (10.2)	
All others^2^	369 (12.0)	96 (12.1)	67 (16.5)	28 (7.3)	
Maternal age at delivery	28.55 (6.6)	28.04 (6.8)	30.15 (6.56)	25.76 (6.3)	***
Maternal age in March 2020	41.54 (7.7)	40.6 (8.0)	42.36 (7.8)	38.72 (7.8)	***
Maternal birthplace					N/A
Outside US	1,841 (59.9)	407 (51.1)	407 (100)	0 (0.0)	
US	1,191 (38.8)	382 (48.0)	0 (0.0)	382 (100.0)	
NA	41 (1.3)	7 (0.9)	0 (0.0)	0 (0.0)	
Highest level of education					***
Less than high school	859 (28.0)	261 (32.8)	145 (35.6)	114 (29.8)	
High school degree	1757 (57.2)	465 (58.4)	213 (52.3)	249 (65.2)	
College degree	425 (13.8)	60 (7.5)	42 (10.3)	17 (4.5)	
NA	32 (1.0)	10 (1.3)	7 (1.7)	2 (0.5)	
Household income at delivery					**
<$15,000	812 (26.4)	246 (30.9)	104 (25.6)	140 (36.6)	
$15,000–$30,000	520 (16.9)	127 (16.0)	79 (19.4)	48 (12.6)	
$30,000–$60,000	256 (8.3)	59 (7.4)	27 (6.6)	32 (8.4)	
$60,000+	108 (3.5)	13 (1.6)	5 (1.2)	8 (2.1)	
Don’t know	1040 (33.8)	262 (32.9)	140 (34.4)	121 (31.7)	
NA	337 (11.0)	89 (11.2)	52 (12.8)	33 (8.6)	
Maternal clinical characteristics					
Maternal BMI category at delivery				*
Overweight/obese	1475 (48.0)	411 (51.6)	211 (51.8)	198 (51.8)	
Underweight/normal	1424 (46.3)	334 (42.0)	163 (40.0)	169 (44.2)	
NA	174 (5.7)	51 (6.4)	33 (8.1)	15 (3.9)	
Maternal chronic hypertension					NS
No	2854 (92.9)	741 (93.1)	381 (93.6)	355 (92.9)	
Yes	204 (6.6)	48 (6.0)	24 (5.9)	24 (6.3)	
NA	15 (0.5)	7 (0.9)	2 (0.5)	3 (0.8)	
Maternal PEH					NS
0	2699 (87.8)	690 (86.7)	361 (88.7)	324 (84.8)	
1	346 (11.3)	97 (12.2)	44 (10.8)	53 (13.9)	
NA	28 (0.9)	9 (1.1)	2 (0.5)	5 (1.3)	
Maternal diabetes					NS
GDM or DM	369 (12.0)	101 (12.7)	60 (14.7)	41 (10.7)	
No	2697 (87.8)	692 (86.9)	347 (85.3)	340 (89.0)	
NA	7 (0.2)	3 (0.4)	0 (0.0)	1 (0.3)	
Maternal cardiometabolic disorders^3^					NS
0	1,788 (58.2)	421 (52.9)	210 (51.6)	207 (54.2)	
1	771 (25.1)	225 (28.3)	116 (28.5)	109 (28.5)	
2	265 (8.6)	77 (9.7)	36 (8.8)	41 (10.7)	
3	53 (1.7)	13 (1.6)	9 (2.2)	4 (1.0)	
N/A	196 (6.4)	60 (7.5)	36 (8.8)	21 (5.5)	
Maternal smoking status					***
Continuous	343 (11.2)	114 (14.3)	12 (2.9)	100 (26.2)	
Never	2,471 (80.4)	606 (76.1)	386 (94.8)	217 (56.8)	
Quitter	227 (7.4)	71 (8.9)	6 (1.5)	65 (17.0)	
N/A	32 (1.0)	5 (0.6)	3 (0.7)	0 (0.0)	

^1^ *P*-value reflects birthplace-stratified comparison among ED users.

^2^ “All others” includes Asian, Pacific Islander, mixed, and other.

^3^ Maternal cardiometabolic disorders include chronic hypertension or preeclampsia, diabetes, and obesity.

*ED*, emergency department; *BBC*, Boston Birth Cohort; *BMI*, body mass index; *PEH*, preecampsia, eclampsia and/or HELLP Syndrome; *GDM*, gestational diabetes; *DM*, diabetes mellitus; *NA*, not available.

**Table 2.** Number of emergency department encounters in 2019 and 2020 among US and foreign-born mothers (column percentages in parentheses).

# of encounters	2019	2020	Total
US born	843 (61.3%)	624 (63.9%)	1467 (62.4%)
Foreign born	526 (38.2%)	347 (35.6%)	873 (37.1%)
N/A^1^	7 (0.5%)	5 (0.5%)	12 (0.5%)
Total	1376	976	2,352

A chi-square test of independence revealed no statistically significant relationship between place of birth and year (P = 0.20).

^1^ N/A refers to individuals for whom information regarding birthplace was not available.

**Table 3.** International Classification of Diseases, 10^th^ Revision, systems categories of primary diagnoses among mothers who visited the emergency department, stratified by year.

	All mothers			Foreign-born mothers			US-born mothers		
ICD-10 systems diagnosis categories	2019 (n)	2020 (n)	% change ‘19–‘20	2019 (n)	2020 (n)	% change ‘19–‘20	2019 (n)	2020 (n)	% change ‘19–‘20
**Certain infectious and parasitic diseases**	**32**	**61**	**90.6**	**13**	**37**	**184.6**	**19**	**24**	**26.3**
Complications of pregnancy, childbirth, and the puerperium	78	34	−56.4	38	16	−57.9	40	18	−55.0
Diseases of the blood and blood-forming organs and certain disorders involving the immune mechanism	212	127	−40.1	2	1	−50.0	210	126	−40.0
Diseases of the circulatory system	67	43	−35.82	32	18	−43.75	35	25	−28.6
**Diseases of the digestive system**	**131**	**98**	**−25.2**	**63**	**28**	**−55.6**	**67**	**69**	**3.0**
Diseases of the ear and mastoid process	10	7	−30.0	5	1	−80.0	5	6	20.0
Diseases of the eye and adnexa	22	13	−40.9	4	8	100.0	18	5	−72.2
Diseases of the genitourinary system	105	80	−23.8	47	30	−36.2	58	50	−13.8
Diseases of the musculoskeletal system and connective tissue	165	96	−41.8	72	41	−43.1	89	54	−39.3
Diseases of the nervous system	9	9	0.0	2	4	100.0	7	5	−28.6
Diseases of the respiratory system	92	49	−46.7	36	21	−41.7	55	28	−49.1
Diseases of the skin and subcutaneous tissue	101	67	−33.7	48	38	−20.8	53	29	−45.3
Endocrine, nutritional, and metabolic diseases	11	13	18.12	8	6	−25.0	3	7	133.3
External causes of morbidity	47	36	−23.4	20	16	−20.0	27	19	−29.6
Factors influencing health status and contact with health services	9	16	77.8	4	5	25.0	5	11	120.0
Injury, poisoning, and certain other consequences of external causes	65	42	−35.4	30	17	−43.3	35	23	−34.3
**Mental, behavioral, and neurodevelopmental disorders**	**44**	**62**	**40.9**	**12**	**8**	**−33.3**	**32**	**54**	**68.8**
Neoplasms	4	1	−75.0	0	1	NA	4	0	−100.0
Symptoms, signs, and abnormal clinical and laboratory findings, not elsewhere classified	163	119	−27.0	88	50	−43.18	74	69	−6.8
Un-codable	9	3	−66.7	2	1	−50.00	7	2	−71.4
Total	1376	976	**−29.1**	526	347	**−34.03**	843	624	**−26.0**

Categories with divergent utilization patterns between the overall cohort, foreign-born mothers, and/or US-born mothers are bolded.

Categories with positive percent increases from 2019 to 2020 and with an n > 10 are also bolded.

*ICD-10*, International Classification of Diseases, 10^th^ Rev.

**Figure 2.**
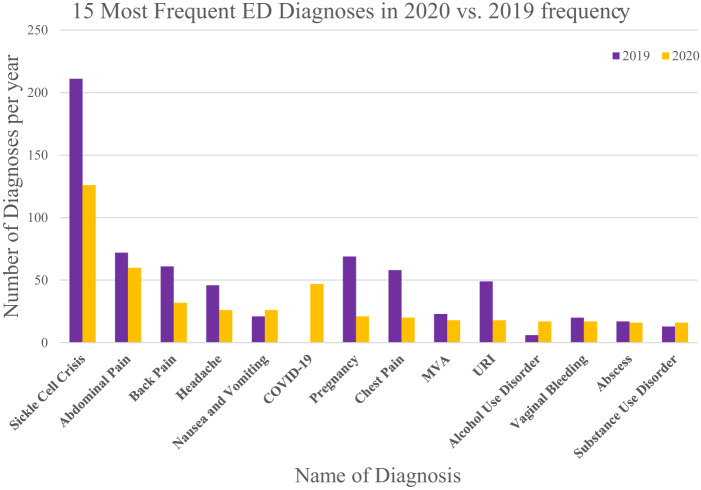
Most frequent emergency department diagnoses among study mothers in 2020 shown with their frequency in 2019.

We categorized ED visit diagnoses from 2019 and 2020 using the ICD-10-CM Tabular List of Diseases and Injuries. Visit diagnoses that could not be characterized into any of the ICD-10-CM systems categories were listed as un-codable. We tabulated the frequency of each category and calculated the percentage change between 2019 and 2020; this was done for both foreign- and US-born subgroups (Table 3). The visit categories that showed a positive percentage increase from 2019 to 2020 (with cell count N > 10) are bolded. Visit categories that displayed a divergent utilization pattern between the study cohort—US- and/or foreign-born mothers—were also bolded. We calculated the frequency of the top 14 diagnoses and analyzed the percentage change using chi-square analysis. The Bonferroni correction method was used to lower the significance threshold due to multiple comparisons. We also compared the top 15 ED visit diagnoses, as well as top mental health diagnoses, between 2019 and 2020 and graphically present them (Figure 2). For all analyses, *P* < 0.05 was considered to be statistically significant.

## RESULTS

### ED Utilization

A sensitivity analysis of ED vs. non-ED users found significant differences with respect to maternal birthplace, race, education, and smoking status (Table 1; Supplemental Table 4). Of the 3,073 mothers enrolled in the full maternal BBC cohort, almost 60% reported being born outside the US (1,841/3,073). Of the 796 in our sample, 51.1% (407/796) of mothers reported being foreign-born (Table 1). Almost 65% of our study sample participants identified as Black, while 19.1% identified as Hispanic. In the full maternal cohort, 58.1% and 22.3% of mothers identified as Black and Hispanic, respectively (Table 1). Only 7.5% of mothers in our study sample reported having a college degree compared to 13.8% in the full maternal cohort. A larger majority (30.9%) of our study sample participants reported a household income of <$15,000 compared to 26% of mothers in the full cohort. Most mothers in our study sample reported never having been smokers (76.1%), with just over 50% of mothers being overweight/obese.

Foreign-born mothers made up over 50% of participants in the study cohort; however they only accounted for 38.2% of ED encounters in 2019 and 35.6% in 2020. Mothers born in the US represented a higher percentage (63.9%) of ED encounters in 2020 compared to 2019 (61.3%), while the proportion of ED encounters completed by foreign-born mothers decreased by 3% (Table 2).

Figure 1 depicts the COVID-19 timeline overlayed with the change in ED visit encounters from 2019 to 2020 for the study cohort—foreign-born mothers and US-born mothers. Across all groups, the largest decline in ED visits was observed in March of 2020 when the stay-at-home order was issued.^21^ Visit frequency slowly increased (without ever reaching pre-pandemic rates) over the following months up until August, when ED visits declined in conjunction with rising COVID-19 cases.^21^ There were a total of 1,376 visit diagnoses in 2019 and 976 in 2020, an absolute decrease of 29.1%. A greater decrease was observed among foreign-born mothers (34.0%) compared to US-born mothers (26.0%), despite foreign-born mothers making up a smaller proportion of ED visits at baseline (Table 2, Figure 1).

Even when controlling for other variables, the results from the negative binomial regression (Supplemental Figure 1, Supplemental Table 3) indicate the decrease in ED visits from 2019 to 2020 was significant (−0.4−1 = 29.7%). The model showed that immigrant status, race/ethnicity, level of education, and low-income are significant predictors of ED visits: foreign-born mothers experienced a 49.4% reduction in utilization rate compared to US-born mothers. Black and Hispanic mothers experienced a 158.1% and 100.7% increase in utilization rate, respectively, compared to White mothers. Mothers with a high school degree and less than high school education had a 77.0% and 142.2% increase in utilization rates, respectively, compared to mothers with a college degree;. Mothers with an annual household income <$60,000 had a 20.6% increase in utilization rate compared to those with incomes >$60,000 per year, all else being equal.

Although nearly significant (*P* = 0.10), the magnitude of the interaction between pandemic indicator by immigrant status in Model 2 suggests there may be a difference in the reduction of ED visits from 2019 to 2020 between foreign- and US-born mothers. The Supplementary Figure 1 shows that the marginal effect of immigrant status is nearly significant for Blacks; ie, immigrant Blacks had a greater reduction in ED visits from 2019 to 2020 than their native-born counterparts.

### Disease-specific Diagnoses in the Emergency Department

We characterized ED visit diagnoses for the study cohort, US- and foreign-born mothers, into one of 15 ICD-10-CM system categories represented in Table 3. Although baseline numbers within each category were small, visits with a large increase or a divergent pattern between the study cohort (n > 10) were bolded. The most common system diagnosis category in both 2019 and 2020 was “diseases of the blood and blood-forming organs and certain disorders involving the immune mechanism” with a frequency of 15.4% (212/1,376) and 13.0% (127/976) in 2019 and 2020, respectively, with US-born mothers making up almost 100% of those visits (210/212, 126/127). While there was an overall decrease from 2019 to 2020, visits for sickle cell crisis were the most common diagnosis within that category both before and during the pandemic (Figure 2).

**Figure 3.**
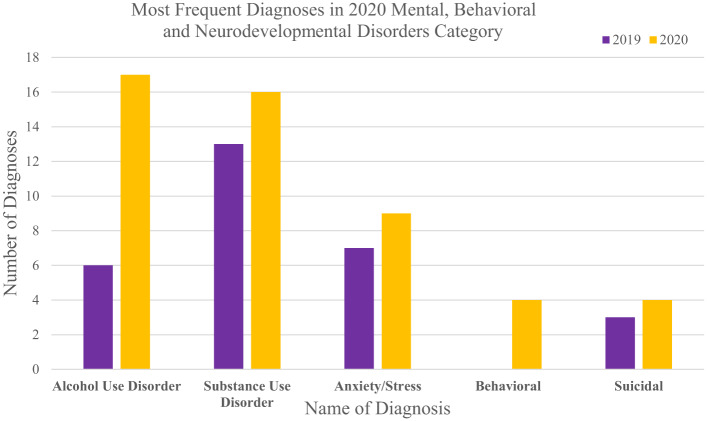
Top five most frequent diagnoses among all mothers in the study, foreign-born and US-born mothers in 2020 within the mental, behavioral, and neurodevelopmental disorders category, as defined by the International Classification of Diseases coding scheme and displayed next to their frequency in 2019.

Within the “certain infectious and parasitic diseases” category, there was a meaningful increase in visits between 2019 and 2020 of 90.7% (*P* = 0.01, Supplemental Table 5). This included visit diagnoses for COVID-19, of which there were zero visits in 2019, but they accounted for 77% of visit diagnoses within that category in 2020 (47/61). Interestingly, the number of visit diagnoses within the “mental, behavioral and neurodevelopmental disorders” category showed a noteworthy increase of 40.9% from 2019 to 2020; however, this was not statistically significant (*P* = 0.10, Supplemental Table 6). This was solely driven by US-born mothers, where there was a 68.8% increase in visits; among foreign-born mothers there was a 33.3% decrease. For the study cohort within this category, ED visits for substance use disorder were the most common in 2019 (13/44) with a modest increase in visits by 23.1% in 2020, while not statistically significant (*P* = 0.20, Figure 3, Supplemental Table 7 and 8). There were zero visits under the behavioral category (which includes diagnoses related to eating and sleep disorders) in 2019; this number increased to four in 2020. Notably, the largest increase of 183.3% was seen in visit diagnoses for alcohol use disorder (*P* = 0.003, Figure 3, Supplemental Table 7 and 8).

## DISCUSSION

Our study demonstrates that the ED continues to serve as a safety net for minority mothers. However, there exists a differential in service needs between our immigrant vs non-immigrant population. In our cohort of urban, low-income, US- and foreign-born mothers, we found a decrease in ED visits of almost 30% from 2019 to 2020. This is less than what has been reported in the current literature, which documented reductions ranging from 40% to over 60%^3^ ^,^ ^22^ ^–^ ^24^; however, these studies examined changes in ED patterns over the first few months of the COVID-19 pandemic as opposed to the entire year. Our results offer a more longitudinal view of changes in ED patterns reflecting the various waves of the COVID-19 pandemic. The parent study also similarly found a reduction in outpatient clinic visits among the same maternal cohort,^8^ consistent with current literature showing declining outpatient visits across the country.^25^ ^–^ ^27^

This raises the question of where the patients who would normally present to the ED went and how they received care. Many institutions expanded their outpatient telemedicine services during the pandemic, which served as one potential avenue to capture missing patients who would otherwise have presented to the ED. However, studies have found that the increase in telemedicine visits was not sufficient to offset the decline observed in the outpatient setting,^25^ ^,^ ^26^ thus challenging the theory as to where ED visits were captured. The observed decrease in total ED visits during the pandemic without a comparable increase in alternative healthcare services could have lingering effects on the continuity and accessibility of healthcare and subsequent health outcomes among this cohort of mothers.

Additionally, we found foreign-born mothers were underrepresented in ED encounters based on their baseline frequency in the full BBC cohort. They also had a greater percentage reduction in ED visits compared to US-born mothers. Results from the negative binomial regression confirmed this trend, again raising the question of where and how this population received care during the pandemic. The BBC participants born inside the US, who are Black or Hispanic, have a high school degree or less, or who are low income have higher rates of ED utilization, regardless of the year. Patterns of ED use among foreign-born individuals vary. Reasons for decreased use could be due to lack of access, fear due to undocumented status, and/or distrust or unfamiliarity with navigating the US healthcare system.^28^ ^,^ ^29^

Despite the overall decrease, the distribution of ED diagnoses did not change dramatically from 2019 to 2020. However, it is important to note that the baseline number of visits for some of the specific ED visit diagnoses was small, which had a larger impact on the relative percentage change. Visits for sickle cell-related crises remained the most common, followed by visits for abdominal pain and back pain in both 2019 and 2020. However, visits related to infectious disease, particularly those for COVID-19, showed an unsurprising increase. While the observed increase among foreign-born mothers was from 13 to 37, it did coincide with the beginning of the first wave of the pandemic, which is consistent with other studies.^24^

Interestingly, our study did demonstrate a trend of increasing ED visits for mental behavioral and neurodevelopmental disorders among mothers, specifically alcohol and substance use disorders. While the observed change was based on relatively smaller baseline numbers, emerging studies have demonstrated a higher incidence of stress, anxiety, and depression among the general population secondary to the COVID-19 pandemic,^5^ ^,^ ^30^ ^,^ ^31^ and women particularly have been reported to be more susceptible to psychological distress.^17^ ^,^ ^32^ Theories for the increased psychological strain are multifaceted; there is the direct impact of the disease itself including fear of infection, disease progression, death, and loss of loved ones.^32^ ^,^ ^33^ There is also the associated stigma, stress related to job loss/security, and issues surrounding prevention, which includes social isolation, school closures, and lack of social support.^32^ Our study reinforces these findings. While our study showed a trend toward increasing visits for substance use disorder and anxiety among mothers, there was a statistically significant increase in visits for alcohol use disorder consistent with other studies,^15^ ^,^ ^34^ with one potential explanation of increased substance and alcohol use as a mechanism for coping with stress and anxiety related to the COVID-19 pandemic.^15^ A recent study by Anderson et al also demonstrated a similar increase in mental health-related ED presentations during the pandemic, particularly among minority populations^35^; however, they did not specifically look at immigration status or a maternal population.

Notably, our study’s observed increase in mental health-related visits was solely driven by US-born mothers, as visits for mental and behavioral disorders for foreign-born mothers were lower at baseline and demonstrated a decline compared to before the pandemic. Similar results were also seen in a study evaluating the effect of COVID-19 on outpatient mental health visits, with US-born mothers showing higher rates of visits.^8^ This is consistent with research that has shown that despite the stress of migration, immigrants tend to experience fewer negative mental and behavioral health outcomes, often termed the “healthy migrant hypothesis,” particularly when they immigrate at younger ages.^18^ ^,^ ^19^ ^,^ ^36^ While there are nuances based on ethnicity and generational status, foreign-born individuals have been found to have lower rates of depressive disorders, anxiety disorders, and substance abuse disorder, with increased social support networks being theorized as one of the reasons for improved mental health outcomes,^18^ which likely contributed to the pattern observed among mothers in our study.

COVID-19 has disproportionately affected people of color,^12^ ^,^ ^14^ and based on our study this pattern remains true as it relates to mental health and substance use disorder. To our knowledge, no previous research has examined changes in ED utilization particularly as it pertains to visits for mental health and substance use disorder, among a minority maternal population. In contrast, a few studies have reported similar disproportionate rates of mental health and substance use disorders among Black and Hispanic patients in relation to the COVID-19 pandemic.^15^ However, they do not differentiate between US- vs foreign-born individuals.

Through an analysis of ED utilization and diagnoses, our study suggests a correlation between the COVID-19 pandemic and increased psychological burden and substance use disorder among minority, low-income, primarily US-born mothers at greater risk for infection and indirect psychological/behavioral disturbance. Furthermore, results from our negative binomial regression model suggest that a larger sample size may help identify the differences in ED utilization among various race/ethnicity and birthplace combinations within our cohort. Further research should be done to determine specific ED patterns related to the immigrant population.

As health systems continue to face additional waves of the COVID-19 pandemic, our study suggests the need to invest in more substance use disorder and mental health resources as we simultaneously expand the capacity to manage those infected with COVID-19. Clinicians should remain vigilant in screening for signs of depression, anxiety, and increased substance dependence in both inpatient and outpatient settings, particularly in low-income and minority populations. The ED should focus efforts on improving the care for a population that has already seen worse outcomes related to COVID-19 and traditionally encounter barriers to receiving treatment for mental health and substance use disorders. This means increasing the availability of substance use disorder counselors, counseling services, social workers, and rehabilitation programs and bolstering systems that refer ED patients to these services. Institutions should also expand the availability of outpatient services to capture these patients upstream of the ED visit to allow for timely intervention and to reduce ED utilization. Additionally, our findings show that the ED remains a critical access point for vulnerable populations, and there are significant differences in service needs among immigrant vs non-immigrant mothers; thus, we as practitioners must take a nuanced approach in both evaluating ED utilization and addressing the needs of these populations in our community.

## LIMITATIONS

Our study focuses explicitly on low-income, urban, minority mothers; thus, our findings may not be generalizable to the larger US population or other populations with different demographic distributions. Nor is it representative of women as a whole, given that our cohort is comprised specifically of women who had given birth to children. Our data overall also relies on small baseline numbers, which had a greater impact on reported percentage changes. Additionally, our intra-pandemic period ran from January to December 2020, including two months that preceded the official declaration of the COVID-19 pandemic by the WHO. Thus, changes in ED utilization in those months may not solely reflect COVID-19. Of note, our study was conducted prior to the onset of widespread vaccination, which also would have impacted ED utilization. Finally, our data relies on the EHR records of study participants who presented to BMC. While unlikely, given that BMC is a comprehensive health system integrated within the Medicaid system, we cannot guarantee that this was the sole facility used by the participants. They may have received services or interacted with the healthcare system outside BMC; therefore, that data would not be represented.

## CONCLUSION

Our study illustrates the importance of the ED as a safety net for healthcare access for minority maternal populations. It supports findings of the psychological burden of the COVID-19 pandemic, showing a need, particularly in minority US-born mothers, for expansion of substance use disorder resources, including inpatient and outpatient treatment centers, rehabilitation programs, and housing support. Our study also shows that immigrant populations have significantly different healthcare service needs and that alternatives for increased care access in the face of pandemics, such as telemedicine, may not be sufficient to appropriately address the needs of those in this community. Therefore, we must take a nuanced approach to better prepare our EDs and communities to handle the consequences of the ongoing pandemic and better plan to face future pandemics.

## Supplementary Information




