# Supplementary material for: Prospective Cohort Study of Emergency Department Visit Frequency and Diagnoses Before and During COVID-19 Pandemic in Urban, Low-Income, US- and Foreign-Born Mothers in Boston, MA
Source: West J Emerg Med. 2023 Nov 8;24(6):1117–27. doi: 10.5811/westjem.59639 (PMC10754186; doi:10.5811/westjem.59639)
Supplement: Supplementary file 1 [file wjem-24-1117-s001.docx]

**Supplemental Materials**

**Supplemental Table 1.** CDC Systems categories of **secondary** diagnoses stratified by year.

| Dx2 Category 3 | 2019 | 2020 | % change |
| --- | --- | --- | --- |
| Certain infectious and parasitic diseases | 26 | 22 | -15.4 |
| Complications of pregnancy, childbirth, and the puerperium | 46 | 12 | -73.9 |
| Diseases of the blood and blood-forming organs and certain disorders involving the immune mechanism | 31 | 3 | -90.3 |
| Diseases of the circulatory system | 44 | 30 | -31.8 |
| Diseases of the digestive system | 67 | 29 | -56.7 |
| Diseases of the ear and mastoid process | 3 | 9 | 200.0 |
| Diseases of the eye and adnexa | 5 | 3 | -40.0 |
| Diseases of the genitourinary system | 53 | 32 | -39.6 |
| Diseases of the musculoskeletal system and connective tissue | 77 | 46 | -40.3 |
| Diseases of the nervous system | 4 | 4 | 0.0 |
| Diseases of the respiratory system | 47 | 32 | -31.9 |
| Diseases of the skin and subcutaneous tissue | 52 | 25 | -51.9 |
| Endocrine, nutritional and metabolic diseases | 3 | 6 | 100.0 |
| External causes of morbidity | 21 | 4 | -81.0 |
| Factors influencing health status and contact with health services | 1 | 1 | 0.0 |
| Injury, poisoning and certain other consequences of external causes | 39 | 11 | -71.8 |
| Mental, Behavioral and Neurodevelopmental disorders | 6 | 3 | -50.0 |
| Mental, Behavioral and Neurodevelopmental Disorders | 30 | 24 | -20.0 |
| Neoplasms | 0 | 1 | 100.0 |
| Symptoms, signs, and abnormal clinical and laboratory findings, not elsewhere classified | 76 | 36 | -52.6 |
| Un-codable | 1 | 2 | 100.0 |
| Total | 632 | 335 | -47.9 |

**Supplemental Table 2.** CDC Systems categories of **tertiary** diagnoses stratified by year.

|  | 2019 | 2020 | % change |
| --- | --- | --- | --- |
| Certain infectious and parasitic diseases | 7 | 2 | -71.4 |
| Complications of pregnancy, childbirth, and the puerperium | 14 | 4 | -71.4 |
| Diseases of the blood and blood-forming organs and certain disorders involving the immune mechanism | 3 | 3 | 0.0 |
| Diseases of the circulatory system | 9 | 2 | -77.8 |
| Diseases of the digestive system | 10 | 2 | -80.0 |
| Diseases of the genitourinary system | 6 | 5 | -16.7 |
| Diseases of the musculoskeletal system and connective tissue | 12 | 4 | -66.7 |
| Diseases of the respiratory system | 7 | 5 | -28.6 |
| Diseases of the skin and subcutaneous tissue | 4 | 1 | -75.0 |
| Endocrine, nutritional and metabolic diseases | 5 | 0 | -100.0 |
| External causes of morbidity | 3 | 1 | -66.7 |
| Injury, poisoning and certain other consequences of external causes | 2 | 1 | -50.0 |
| Mental, Behavioral and Neurodevelopmental disorders | 1 | 1 | 0.0 |
| Mental, Behavioral and Neurodevelopmental Disorders | 2 | 8 | 300.0 |
| Symptoms, signs and abnormal clinical and laboratory findings, not elsewhere classified | 10 | 13 | 30.0 |
| Total | 95 | 52 | -45.3 |

**Supplemental Table 3**. Results from negative binomial regression modeling.

| Variable | Coefficient (Standard Error) | |
| --- | --- | --- |
|  | Model 1: Multivariate | Model 2: Interaction term |
| Pandemic indicator | -0.4*** | -0.3*** |
|  | (0.1) | (0.1) |
| Immigrant status | -0.7*** | -0.601*** |
|  | (0.1) | (0.1) |
| Black | 0.9*** | 1.0*** |
|  | (0.2) | (0.2) |
| Hispanic | 0.7** | 0.7** |
|  | (0.2) | (0.2) |
| Other races | 1.2*** | 1.2*** |
|  | (0.2) | (0.2) |
| High school | 0.6*** | 0.6*** |
|  | (0.2) | (0.2) |
| Lower than high school | 0.9*** | 0.9*** |
|  | (0.2) | (0.2) |
| Low Income | 0.2* | 0.2* |
|  | (0.1) | (0.1) |
| Age at delivery < 35 | 0.1 | 0.1 |
|  | (0.1) | (0.1) |
| Pandemic-period * Outside US | - | -0.2 |
|  | - | (0.1) |
| _cons | -0.5 | -0.5 |
|  | (0.3) | (0.3) |
| Statistics |  |  |
| n | 5384 | 5384 |
| Ave. Group size | 2692 | 2692 |
| Model df | 9 | 10 |
| chi2 | 149.3 | 148.0 |
| p-value | 0.0 | 0.0 |

legend: * p<.05; ** p<.01; *** p<.001


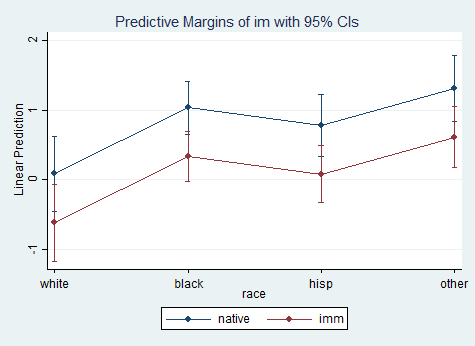


**Supplemental Figure 1**. Margins plot showing Model 1’s predicted values by birthplace and race groups.

**Supplemental Table 4.** Summary of preconception and perinatal characteristics of the participating mothers.

|  |  | ED visit in 2019 and/or 2020 | |  |
| --- | --- | --- | --- | --- |
| Characteristic (% or mean (SD)) | | No | Yes | P value |
| n | | 2277 | 796 |  |
| **Maternal Demographic Characteristics** | |  |  |  |
|  | Maternal Race |  |  | **<0.001** |
|  | Non-Hispanic White | 198 (8.7) | 34 (4.3) |  |
|  | Non-Hispanic Black | 1272 (55.9) | 514 (64.6) |  |
|  | Hispanic | 534 (23.5) | 152 (19.1) |  |
|  | All Others^1^ | 273 (12.0) | 96 (12.1) |  |
|  | Maternal Age at Delivery | 28.72 (6.5) | 28.04 (6.8) | **0.01** |
|  | Maternal Age in March 2020 | 41.85 (7.5) | 40.64 (8.0) | **<0.001** |
|  | Maternal Birthplace |  |  | **<0.001** |
|  | Outside US | 1434 (63.0) | 407 (51.1) |  |
|  | US | 809 (35.5) | 382 (48.0) |  |
|  | NA | 34 (1.5) | 7 (0.9) |  |
|  | Highest level of education |  |  | **<0.001** |
|  | Less than high school | 598 (26.3) | 261 (32.8) |  |
|  | High school degree | 1292 (56.7) | 465 (58.4) |  |
|  | College degree | 365 (16.0) | 60 (7.5) |  |
|  | NA | 22 (1.0) | 10 (1.3) |  |
|  | Household income at delivery |  |  | **0.001** |
|  | <$15,000 | 566 (24.9) | 246 (30.9) |  |
|  | $15,000-$30,000 | 393 (17.3) | 127 (16.0) |  |
|  | $30,000-$60,000 | 197 (8.7) | 59 (7.4) |  |
|  | $60,000+ | 95 (4.2) | 13 (1.6) |  |
|  | Don't know | 778 (34.2) | 262 (32.9) |  |
|  | NA | 248 (10.9) | 89 (11.2) |  |
| **Maternal Clinical Characteristics** | |  |  |  |
|  | Maternal BMI Category at Delivery |  |  | **0.02** |
|  | Overweight/Obese | 1064 (46.7) | 411 (51.6) |  |
|  | Underweight/Normal | 1090 (47.9) | 334 (42.0) |  |
|  | NA | 123 (5.4) | 51 (6.4) |  |
|  | Maternal Chronic Hypertension |  |  | 0.1 |
|  | No | 2113 (92.8) | 741 (93.1) |  |
|  | Yes | 156 (6.9) | 48 (6.0) |  |
|  | NA | 8 (0.4) | 7 (0.9) |  |
|  | Maternal PIH |  |  | 0.5 |
|  | 0 | 2009 (88.2) | 690 (86.7) |  |
|  | 1 | 249 (10.9) | 97 (12.2) |  |
|  | NA | 19 (0.8) | 9 (1.1) |  |
|  | Maternal Diabetes |  |  | 0.5 |
|  | GDM or DM | 268 (11.8) | 101 (12.7) |  |
|  | No | 2005 (88.1) | 692 (86.9) |  |
|  | NA | 4 (0.2) | 3 (0.4) |  |
|  | Maternal Cardiometabolic Disorders^2^ |  |  | **0.01** |
|  | 0 | 1367 (60.0) | 421 (52.9) |  |
|  | 1 | 546 (24.0) | 225 (28.3) |  |
|  | 2 | 188 (8.3) | 77 (9.7) |  |
|  | 3 | 40 (1.8) | 13 (1.6) |  |
|  | NA | 136 (6.0) | 60 (7.5) |  |
|  | Maternal Smoking Status |  |  | **0.001** |
|  | Continuous | 229 (10.1) | 114 (14.3) |  |
|  | Never | 1865 (81.9) | 606 (76.1) |  |
|  | Quitter | 156 (6.9) | 71 (8.9) |  |
|  | NA | 27 (1.2) | 5 (0.6) |  |

^1^“All Others” includes Asian, Pacific Islander, Mixed, and Other

^2^Maternal Cardiometabolic Disorders includes chronic hypertension or preeclampsia, diabetes, and obesity

**Supplemental Table 5.** Number of ED encounters in “Certain infectious and parasitic diseases” category in 2019 and 2020 among US and foreign-born mothers with column percentages in parentheses.

| **# of Encounters** | 2019 | 2020 | Total |
| --- | --- | --- | --- |
| US born | 19 (40.6%) | 24 (60.7%) | 43 (46.2%) |
| Foreign born | 13 (59.4%) | 37 (39.3%) | 50 (53.8%) |
| Total | 32 | 61 | 93 |

A Chi-Square test of independence revealed no statistically significant relationship between place of birth.

**Supplemental Table 6.** Number of ED encounters in “Mental, Behavioral and Neurodevelopmental Disorders” category in 2019 and 2020 among US and foreign-born mothers with column percentages in parentheses.

| **# of Encounters** | 2019 | 2020 | Total |
| --- | --- | --- | --- |
| US born | 32 (72.7%) | 54 (87.1%) | 86 (81.1%) |
| Foreign born | 12 (27.3%) | 8 (12.9%) | 20 (18.9%) |
| Total | 44 | 62 | 106 |

A Chi-Square test of independence revealed no statistically significant relationship between place of birth and year among mental and behavioral diagnoses (P = 0.1).

**Supplemental Table 7**. Top 14 diagnoses in 2020 among mothers and % change from 2019 to 2020.

| Most common diagnoses | 2019 | 2020 | % change | p-value |
| --- | --- | --- | --- | --- |
| Sickle Cell Crisis | 211 | 126 | -40.1 | 0.1 |
| Abdominal Pain | 72 | 60 | -16.7 | 0.4 |
| Back Pain | 61 | 32 | -47.5 | 0.4 |
| Headache | 46 | 26 | -43.4 | 0.4 |
| Nausea & Vomiting | 21 | 26 | 23.8 | 0.1 |
| **COVID-19^1^** | **0** | **47** | **Increase from 0** | **<0.0001** |
| **Pregnancy** | **69** | **21** | **-69.6** | **0.0005** |
| Chest Pain | 58 | 20 | -65.5 | 0.006 |
| MVA | 23 | 18 | -21.7 | 0.9 |
| URI | 49 | 18 | -63.3 | 0.02 |
| **Alcohol Abuse** | **6** | **17** | **183.3** | **0.003** |
| Vaginal Bleeding | 20 | 17 | -15.0 | 0.7 |
| Abscess | 17 | 16 | -5.9 | 0.5 |
| Substance Abuse | 13 | 16 | 23.1 | 0.2 |
| Total ED diagnoses | 1376 | 976 | -29.1 |  |

^1^Includes exposure and diagnosis

URI, Upper Respiratory tract Infection; MVA, Motor Vehicle Accident

The top 14 diagnoses make up approximately 50% of the total diagnoses. P-values calculated using Chi-square tests. Bonferroni correction method was used to lower the significance threshold to p < 0.004.

**Supplemental Table 8.** Top five diagnoses among all study mothers in Mental, Behavioral and Neurodevelopmental Disorders diagnosis category.

|  | 2019 | 2020 | % change |
| --- | --- | --- | --- |
| Alcohol Abuse | 6 | 17 | 183.3 |
| Substance Abuse | 13 | 16 | 23.1 |
| Anxiety/Stress | 7 | 9 | 28.6 |
| Behavioral | 0 | 4 | Increase from 0 |
| Suicidal | 3 | 4 | 33.3 |
|  |  |  |  |
